# The Resistance of Human APOBEC3H to HIV-1 NL4-3 Molecular Clone Is Determined by a Single Amino Acid in Vif

**DOI:** 10.1371/journal.pone.0057744

**Published:** 2013-02-28

**Authors:** Marcel Ooms, Michael Letko, Mawuena Binka, Viviana Simon

**Affiliations:** 1 Department of Microbiology, Mount Sinai School of Medicine, New York, New York, United States of America; 2 Global Health and Emerging Pathogens Institute, Mount Sinai School of Medicine, New York, New York, United States of America; 3 Division of Infectious Diseases, Department of Medicine, Mount Sinai School of Medicine, New York, New York, United States of America; Centro de Biología Molecular Severo Ochoa (CSIC-UAM), Spain

## Abstract

Some human APOBEC3 cytidine deaminases have antiviral activity against HIV-1 and other retroviruses. The single deaminase domain APOBEC3H (A3H) enzyme is highly polymorphic and multiple A3H haplotypes have been identified. A3H haplotype II (A3H-hapII) possesses the strongest activity against HIV-1. There remains, however, uncertainty regarding the extent to which A3H-hapII is sensitive to HIV-1 Vif mediated degradation. We tested, therefore, the two different reference Vif proteins widely used in previous studies. We show that A3H-hapII is resistant to NL4-3 Vif while it is efficiently degraded by LAI Vif. Co-immunoprecipitation assays demonstrate that LAI Vif, but not NL4-3 Vif associates with A3H-hapII. Chimeras between NL4-3 and LAI Vif identify the amino acid responsible for the differential degradation activity: A histidine at position 48 in Vif confers activity against A3H-hapII, while an asparagine abolishes its anti-A3H activity. Furthermore, the amino acid identity at position 48 only affects the degradation of A3H-hapII, whereas recognition of and activity against human A3D, A3F and A3G are only minimally affected. NL4-3 encoding 48H replicates better than NL4-3 WT (48N) in T cell-lines stably expressing A3H hapII, whereas there is no fitness difference in the absence of APOBEC3. These studies provide an explanation for the conflicting reports regarding A3H resistance to Vif mediated degradation.

## Introduction

The human APOBEC3 family consists of seven deaminase proteins (A3A to A3H) that can restrict HIV-1 in cell culture [1], [2]. They exert their activity by deaminating single stranded viral cDNA during reverse transcription resulting in numerous G-to-A mutations in the provirus [3], [4], [5]. HIV-1 Vif can counteract the restrictive activity of several APOBEC3 proteins by mediating their proteasomal degradation [6], [7].

APOBEC3H (A3H), one of the single deaminase domain enzymes, was initially found to lack anti-HIV-1 activity due to its low protein stability [8], [9], [10]. However, several studies showed in 2008 that, in addition to the unstable reference wild-type A3H protein (WT, A3H-hapI), multiple other A3H haplotypes exist [11], [12], [13]. In contrast to A3H-hapI, A3H-hapII, hapIII, hapIV and hapV are stably expressed and can potently restrict HIV-1. A3H-hapII (RDD) differs at three amino acid positions (G105R, K121D and E178D) from A3H-hapI (GKE), but only the arginine (R) at position 105 is responsible for the increased protein stability [11], [12]. The allelic frequency of the active A3H-hapII is high in African and low in European and Asian populations [11], [14].

While there is good agreement on the potent antiviral activity of A3H-hapII, there remains uncertainty regarding its susceptibility to HIV-1 mediated proteosomal degradation. Indeed, multiple studies looking at the sensitivity of A3H-hapII to HIV-1 Vif revealed discrepant results; In some reports A3H-hapII expression was unaffected by HIV-1 Vif co-expression and no difference in restriction was observed between HIV-1 WT and HIV-1 lacking a functional Vif (HIV-1 ΔVif) when produced in the presence of A3H-hapII [12], [14], [15], [16], [17]. Other studies showed, however, efficient degradation of A3H-hapII by Vif and, consequently, infectivity of HIV-1 ΔVif but not of HIV-1 WT was reduced in the presence of A3H-hapII [11], [17], [18], [19], [20]. Interestingly, the studies that observed sensitivity of A3H-hapII to HIV-1 Vif also found that it was solely determined by the nature of the amino acid located at A3H position 121 [18], [20]. Replacing the aspartic acid (D) in A3H-hapII with a lysine (K, RDD to RKD) at this position resulted in a Vif-resistant protein that would restrict HIV-1 in a Vif-independent manner [18], [20].

The commonly used HIV molecular clones, NL4-3 and LAI, differ in their Vif coding region at multiple positions. We hypothesized that these Vif differences affect the resistance to A3H-hapII. Most of the studies that reported A3H-hapII being sensitive to Vif used the HIV-1 LAI clone [11], [18], [19], whereas the studies that observed A3H-hapII resisting Vif degradation used HIV-1 NL4-3 [12], [14], [15], [16]. Only one study made a direct comparison between NL4-3 and LAI Vif variants and found, indeed, a clear difference between their activities towards A3H hapII [17]. Interestingly, one report stated that NL4-3 Vif efficiently degraded A3H-hapII and thereby rescued HIV-1 infectivity [20]. However, the A3H variant used in that study encoded a glutamic acid (E) instead of an aspartic acid (D) at position 121. 121D in A3H-hapII is the result of two mutations at the first and third position of the same codon (AAG = G to GAC = D), whereas the SNP database lists the polymorphisms separately (AAC = N; GAG = E), resulting in distinct residues (121E and 121N, respectively). These two SNPs are believed to be linked and, thus, natural A3H transcript variants were always found to encode an aspartic acid at that position [11], [12], [14].

The aim of this study was to clarify the discrepancies regarding A3H resistance to HIV-1 Vif. We systematically assessed the properties of NL4-3 and LAI Vif and show that A3H-hapII is efficiently counteracted by LAI Vif but not to NL4-3 Vif. This difference in A3H recognition can be attributed to a single amino acid difference at Vif position 48. Exchanging the respective amino acids at position 48 between NL4-3 and LAI Vif reversed the degradation phenotype against A3H-hapII. Furthermore, we show using single cycle and multiple round infections that Vif position 48 is highly specific for A3H-hapII degradation as the neutralization of other APOBEC3 members was unaffected.

## Results

### A3H Sensitivity to NL4-3 and LAI Vif

**Figure 1:**
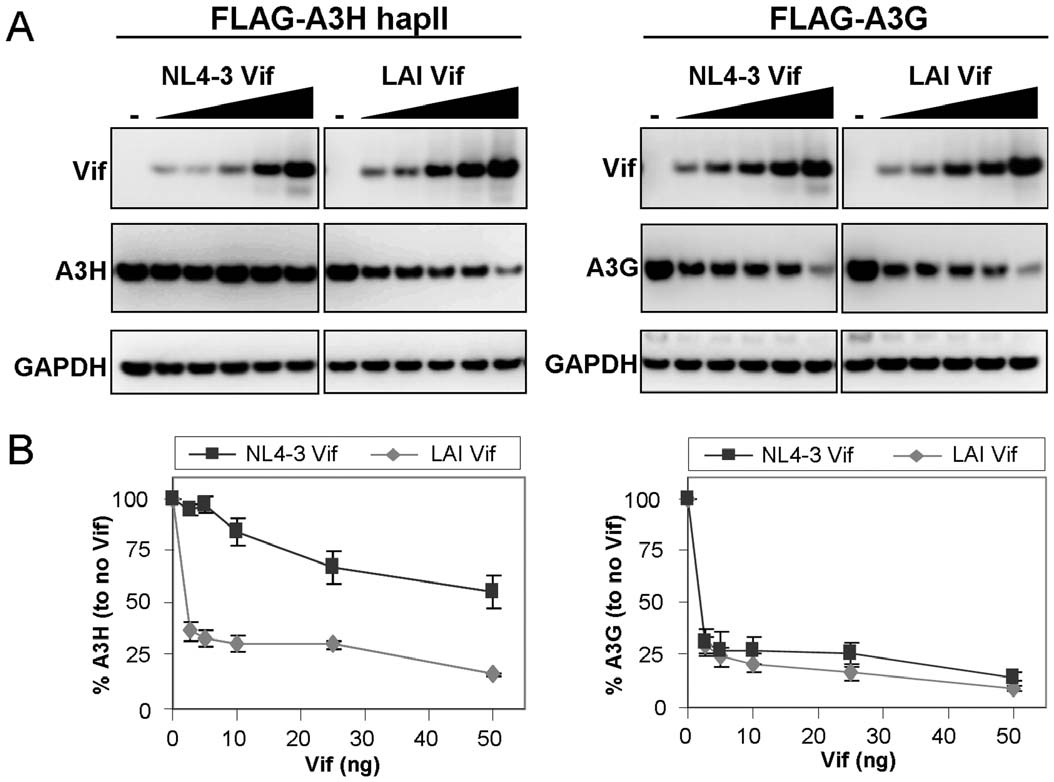
APOBEC3H sensitivity to NL4-3 and LAI Vif. (**A**) Increasing amounts of NL4-3 or LAI Vif expression plasmids (0, 2.5, 5, 10, 25 and 50 ng) were cotransfected with 100 ng of FLAG-tagged A3H-hapII or A3G in 293T cells. Two days post transfection cells were lysed and analyzed by western blot. (**B**) A3H-hapII and A3G expression from (A) were quantified by measuring non-saturated signals using the Fujifilm Intelligent Lightbox LAS-3000 instrument and Image Reader LAS-3000 software. Signals were normalized by setting both A3H-hapII and A3G expression levels without Vif at 100%. Error bars represent standard deviation from three independent experiments.

To test whether the difference in Vif sensitivity of A3H-hapII is caused by the different Vif variants, we first determined the efficiency of Vif-mediated A3H-hapII degradation in the producer cell. We co-transfected increasing amounts of NL4-3 or LAI Vif pCRV1 expression plasmids with A3H-hapII or A3G expression plasmids. A3H and A3G protein levels were analyzed two days after transfection by western blot (Figure 1A) and the intensities of the unsaturated A3H signals were quantified (Figure 1B). NL4-3 and LAI Vif showed different effects on A3H expression levels despite being expressed to similar levels (Figure 1A). NL4-3 Vif, even at high expression levels, only modestly degraded A3H-hapII, whereas small quantities of LAI Vif were sufficient for efficient A3H-hapII degradation (Figure 1B). Both Vif variants degraded A3G with similar efficiency indicating that the two Vif variants are functionally comparable with respect to A3G but specifically differ in their efficiency to degrade A3H-hapII (Figure 1A and 1B).

### A3H-hapII and A3G Restriction of Vif Proficient and Vif Deficient HIV-1 NL4-3 and LAI

**Figure 2:**
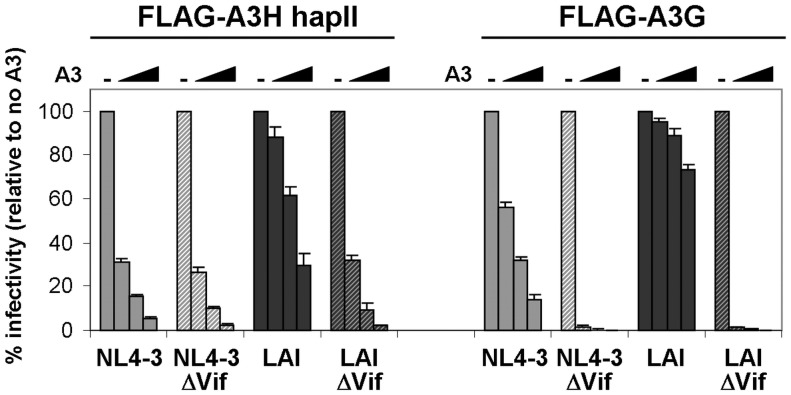
A3H-hapII and A3G restriction of Vif proficient and Vif deficient HIV-1 NL4-3 and LAI molecular clones. Increasing amounts of A3H-hapII or A3G (0, 25, 50 and 100 ng) were cotransfected with the different HIV molecular clones (500 ng) in 293T cells. Two days post transfection cleared supernatants were used to infect TZM-bl reporter cells and β-Galactosidase was measured two days later. One representative experiment of three consisting of triplicate transfections is shown. Error bars represent standard deviations from triplicate transfections.

Next, we tested whether Vif expressed *in cis* from their respective full-length HIV-1 molecular clone would counteract A3H-hapII restriction. Increasing amounts of A3H-hapII and A3G were transfected with full-length NL4-3, LAI or their corresponding Vif deleted counterparts (ΔVif). Infectivity was measured by infecting TZM-bl reporter cells [21]. Figure 2 shows that NL4-3 WT and NL4-3 ΔVif were restricted to similar levels by A3H-hapII, whereas LAI WT was resistant to A3H-hapII restriction. In good agreement with numerous reports [reviewed in [22]], A3G potently restricted NL4-3 ΔVif and LAI ΔVif, but the two wild-type viruses counteracted A3G efficiently. Interestingly, NL4-3 WT was more sensitive to A3G than LAI WT, suggesting that LAI Vif also counteracts A3G more efficiently than NL4-3 Vif. These results show that NL4-3 Vif and LAI Vif expressed from full-length HIV-1 both counteracted A3G, but only LAI Vif counteracted A3H-hapII. The infectivity data is well supported by the A3H-hapII and A3G Vif degradation results (Figure 1). Taken together, A3H-hapII is counteracted by LAI Vif, but not by NL4-3 Vif.

### Contribution of the Three A3H-hapII Single Nucleotide Polymorphisms to Vif Resistance

**Figure 3:**
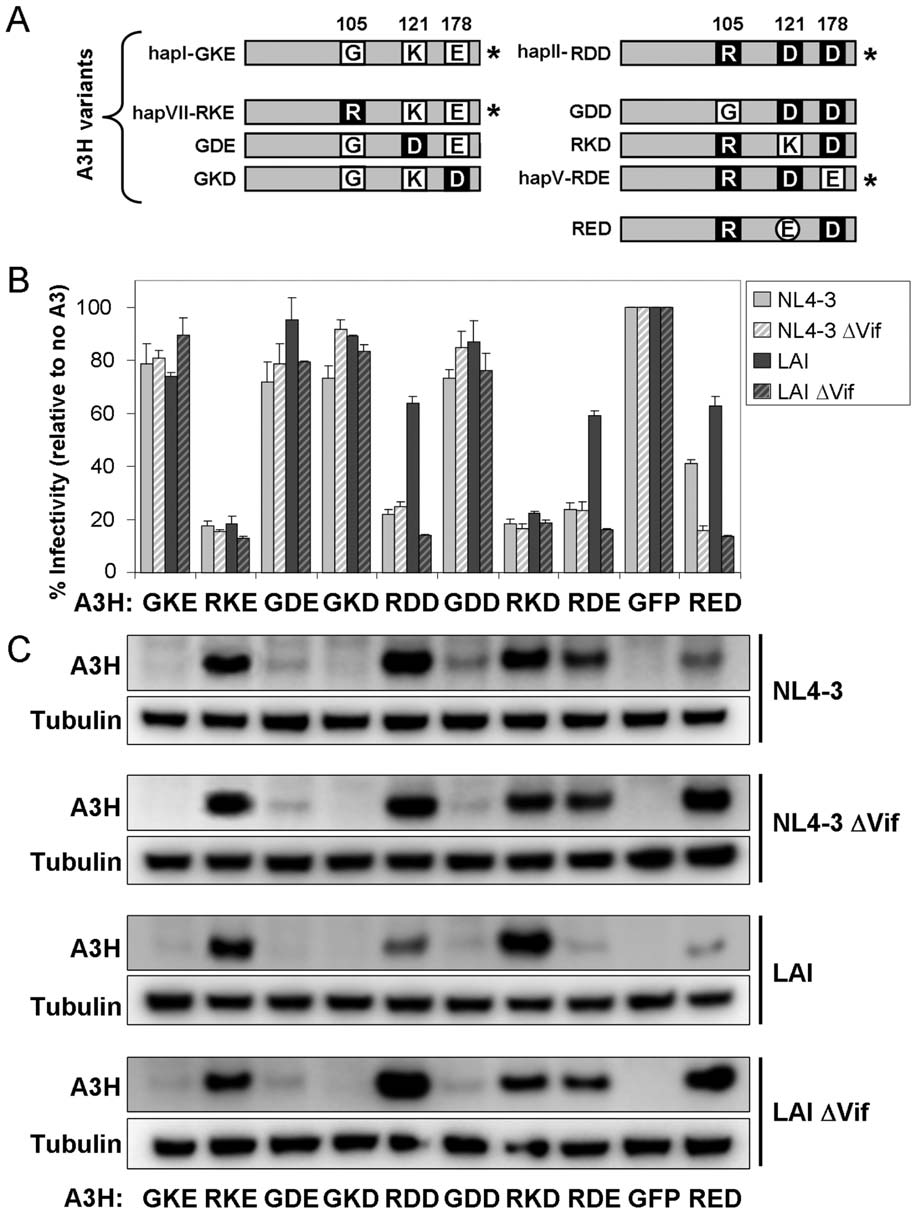
Schematic overview of A3H-hapI, A3H-hapII and mutants and their antiviral activity. (**A**) The respective amino acids at positions 105, 121 and 178 are indicated. Asterisks denote natural variants [11], [14]. (**B**) A3H variants (20 ng) were transfected with the indicated HIV plasmids in 293T cells. Two days later, cleared supernatants were used to infect TZM-bl cells after which β-Galactosidase activity was measured two days later. One representative experiment consisting of triplicate transfections is shown. Error bars represent standard deviations from triplicate transfections. (**C**) 293T cells from (**B**) were lysed and analyzed by western blot for A3H expression (FLAG). Tubulin served as a loading control.

In order to determine the putative contribution of A3H haplotypes to the observed Vif phenotype, we analyzed the impact of the three amino acids differing between A3H-hapI (GKE) and A3H-hapII (RDD) on the restriction of WT NL4-3 and LAI and their Vif deleted counterparts. Previous studies showed that the G105R change promotes protein stability and antiviral activity [11], [12], whereas K121D renders A3H-hapII sensitive to Vif degradation [18]. Additional A3H haplotypes encoding other combinations of these three amino acids (e.g. hapV-RDE, hapVI-GKD and hapVII-RKE) were recently described [14]. A panel of A3H mutants comprising all the possible combinations of the three residue changes were tested for antiviral activity and Vif sensitivity [Figure 3A, [12]]. We also included the artificial A3H-hapII RED variant, which was shown to be sensitive to NL4-3 Vif [20]. We analyzed the effect of these A3H variants on infectivity of NL4-3 and LAI molecular clones with and without Vif. All A3H variants encoding 105G, including A3H-hapI, failed to restrict HIV-1 (A3H-GKE, GDE, GKD, and GDD, Figure 3B). The 105R encoding variants (RKE, RDD, RKD and RDE) restricted NL4-3 WT, NL4-3 ΔVif and LAI ΔVif irrespective of the amino acid at position 121 (Figure 3B). LAI Vif could only efficiently counteract the restriction of the 121D variants (A3H-RDD and RDE), but not that of A3H-hapII encoding 121K (A3H-RKE and RKD, Figure 3B). The A3H RED variant was also sensitive to LAI Vif but, more importantly, also showed some sensitivity to NL4-3 Vif, indicating that the glutamic acid (E) at position 121 renders A3H sensitive to both LAI and NL4-3 Vif (Figure 3B). Western blot analysis of A3H expression in 293T cells showed that all four 105G carrying A3H variants (GKE, GDE, GKD and GDD) were poorly expressed and failed to restrict HIV lacking Vif (Figure 3B and 3C). A3H RKE and RKD variants were resistant to NL4-3 and LAI Vif-mediated degradation. A3H RDD and A3H RDE were both exclusively degraded by LAI Vif, but not by NL4-3 Vif. The artificial A3H RED variant was degraded by both NL4-3 and LAI Vifs. Combined, the A3H degradation results in Figure 3C are in excellent agreement with the restriction patterns depicted in Figure 3B. Taken together, these data confirm that the amino acid at position 121 determines the sensitivity of A3H to degradation by specific Vif variants: An aspartic acid (A3H 121D) mediates A3H sensitivity to LAI Vif [18], a glutamic acid (A3H 121E) renders A3H sensitive to both LAI and NL4-3 Vif and a lysine (121K) results in resistance of A3H to both Vif alleles.

### Association of A3H-hapII with LAI Vif, but not with NL4-3 Vif

**Figure 4:**
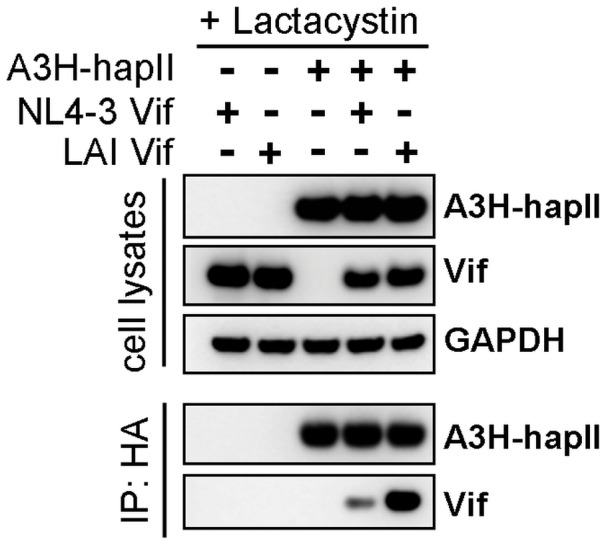
Association of A3H-hapII with LAI Vif, but not with NL4-3 Vif. 5 µg HA-tagged A3H-hapII was co-transfected with 1 µg of the indicated Vif expression plasmids in 293T cells in 10-cm dishes. Cells were treated with clasto-Lactacystin β-lactone (10 µM) for ten hours and cells were lysed in mild lysis buffer 2 days post transfection. Lysates were cleared and incubated with anti-HA tagged beads (Sigma) for one hour at 4°C. Beads were extensively washed with lysis buffer and proteins were eluted by boiling in sample loading buffer. Proteins were analyzed by western blot.

The observation that A3H-hapII is sensitive to LAI Vif but not NL4-3 Vif could be explained by a difference in Vif binding to A3H-hapII. We studied the interaction of A3H-hapII with both Vif variants by co-immunoprecipitation. HA-tagged A3H-hapII was co-transfected with empty pCRV1 plasmids or pCRV1 expressing NL4-3 and LAI Vif in 293T cells. To block A3H degradation by LAI Vif, the proteasome inhibitor, clasto-Lactacystin β-lactone, was added to the transfected HEK 293T cells 24 hours prior to cell lysis. Cleared lysates were incubated with α-HA coated beads and extensively washed with lysis buffer and eluted. Western blot analysis of the cell lysates showed that Vifs and A3H were equally expressed and that A3H-hapII was not degraded by LAI Vif in the presence of proteasome inhibitor (Figure 4). LAI Vif co-precipitated efficiently with A3H-hapII, whereas only very little NL4-3 Vif was bound (Figure 4). Together, this indicates that LAI Vif, but not NL4-3 Vif, can efficiently associate with A3H-hapII. This ability of LAI Vif correlates with its efficiency to degrade and counteract the antiviral activity of A3H-hapII.

### Vif Position 48 Determines A3H hapII Degradation and Rescue of Infectivity

**Figure 5:**
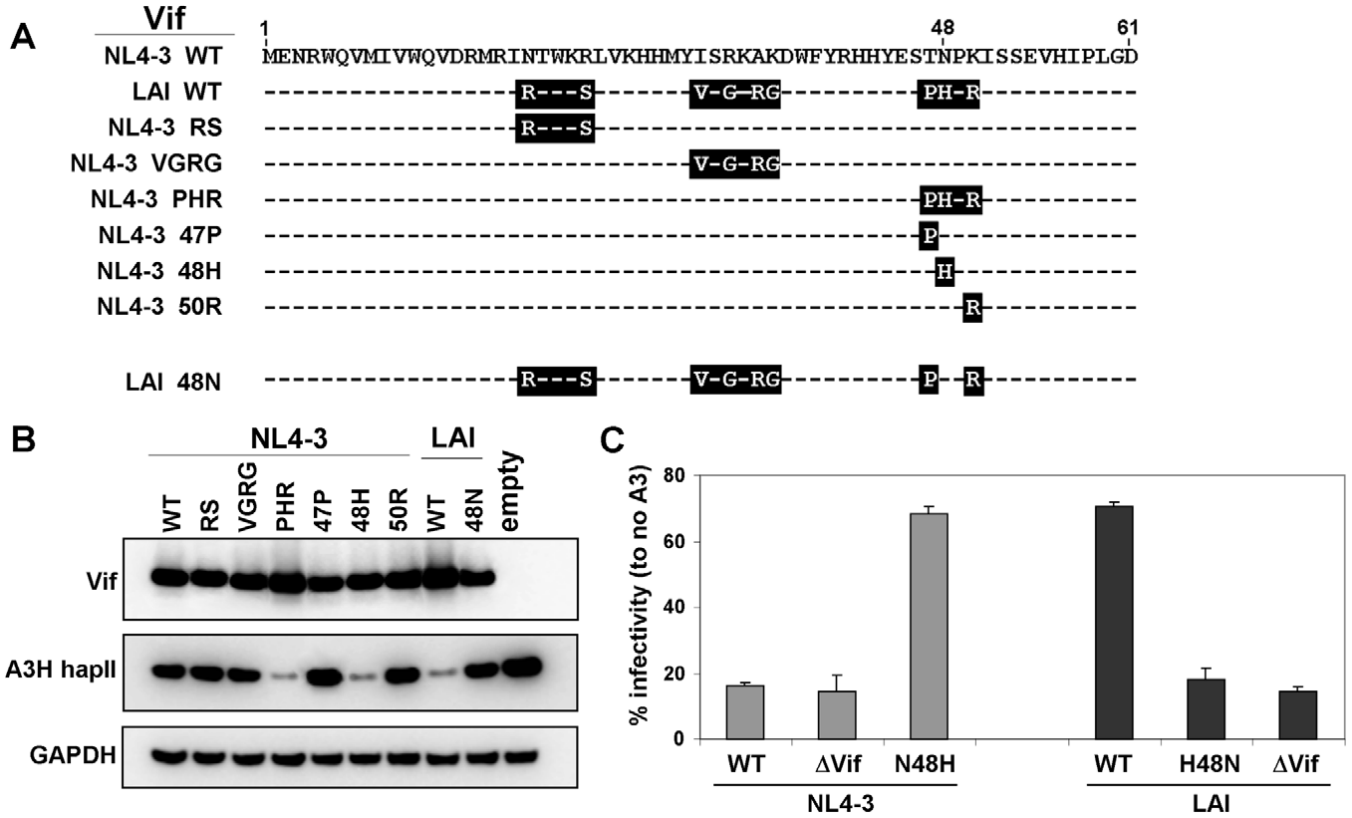
Vif 48H is required for A3H hapII degradation and rescue of infectivity. (**A**) Schematic overview of the first 61 amino acids of NL4-3 and LAI Vif. Three regions that are different between NL4-3 and LAI are indicated by black boxes. (**B**) 50 ng NL4-3 or LAI or mutant Vif expression plasmids was co-transfected with 100 ng of FLAG-tagged A3H-hapII in 293T cells. Two days post transfection cells were lysed and analyzed by western blot. (**C**) A3H-hapII restriction of Vif proficient mutant Vif and Vif deficient HIV-1 NL4-3 and LAI. 50 ng A3H-hapII was co-transfected with the different indicated HIV molecular clones (500 ng) in 293T cells. Two days post infection cleared supernatants were used to infect TZM-bl reporter cells and β-Galactosidase was measured two days later. One representative experiment consisting of triplicate transfections is shown. Error bars represent standard deviations from triplicate transfections.

NL4-3 and LAI Vifs only differ at nine positions in the N-terminal region (see Figure 5A), which is the portion of Vif critical for interaction with several APOBEC3 proteins. In order to pinpoint the differential anti-A3H activity at a single residue level, we generated a set of Vif chimeras in which LAI Vif amino acids were introduced into NL4-3 Vif (Figure 5A). The A3H-hapII degradation efficiency in the presence of the WT NL4-3, WT LAI and the chimeras was analyzed by western blot. The introduction of either LAI amino acids RS or VGRG into NL4-3 Vif did not results in an increase in activity, indicating that these regions are dispensable for specific A3H-hapII recognition (Figure 5B). However, a NL4-3 Vif variant with amino acids PHR efficiently degraded A3H-hapII. Additional single amino acid changes within the PHR stretch revealed that the introduction of a histidine at position 48 was sufficient to fully confer activity towards A3H-hapII. Indeed, the reverse change in LAI Vif, H48N, resulted in a complete loss of A3H-hapII degradation activity (Figure 5B). We next replaced the Vif position 48 in the full-length molecular clones NL4-3 and LAI and tested their infectivity levels in the presence of A3H-hapII (Figure 5C). Both NL4-3 WT and NL4-3 ΔVif were restricted, but the restriction was clearly relieved for NL4-3 N48H. Conversely, LAI WT displayed high infectivity levels, but the H48N change efficiently restricted LAI H48N, similar to LAI ΔVif. In summary, the difference in counteracting A3H-hapII between NL4-3 and LAI Vif can be attributed to a single amino acid at position 48.

### Vif Position 48 is Specific for A3H-hapII Recognition

**Figure 6:**
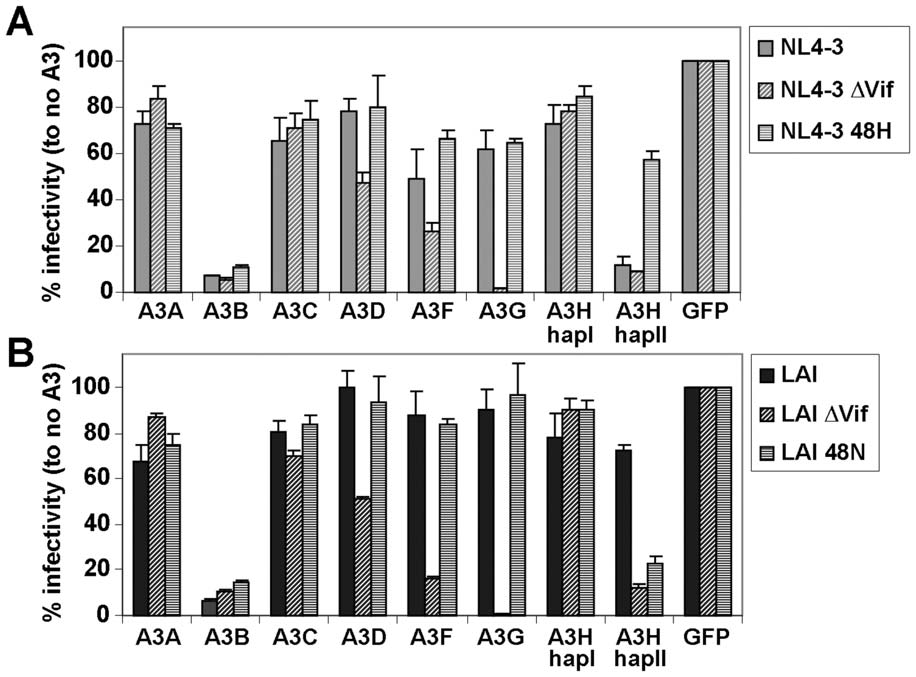
Vif position 48 is specific for A3H-hapII. (**A**) 500 ng NL4-3, NL4-3 ΔVif, NL4-3 48H and (**B**) LAI, LAI ΔVif and LAI 48N was co-transfected with 20 ng of the different HA-tagged APOBEC3 in 293T cells. Two days post infection cleared supernatants were used to infect TZM-bl reporter cells and β-Galactosidase was measured two days later. One representative experiment consisting of triplicate transfections is shown. Error bars represent standard deviations from triplicate transfections.

To test whether Vif position 48 affected the neutralization of other APOBEC3 proteins in addition to A3H-hapII, we analyzed the infectivity levels of WT NL4-3, and LAI as well as the corresponding Vif deleted and 48 substituted molecular clones in the presence of all seven human APOBEC3 members. NL4-3 ΔVif and LAI ΔVif were both restricted by A3B, A3D, A3F, A3G and A3H hapII to varying degrees (Figure 6). The restriction by A3D, A3F and A3G was relieved by all the Vif variants. Both LAI and NL4-3 48H Vif efficiently counteracted A3H-hapII, whereas NL4-3 and LAI 48N showed low infectivity in the presence of A3H-hapII. In these single cycle infectivity experiments, the Vif 48 mutants in both NL4-3 and LAI molecular backgrounds counteracted A3D, A3F and A3G as efficiently as the parental clones indicating that the Vif substitution at position 48 specifically affects A3H recognition without impacting other APOBEC3 proteins.

### NL4-3 Vif 48H Replicates Better than NL4-3 WT in SupT1 Cells Stably Expressing A3H-hapII

**Figure 7:**
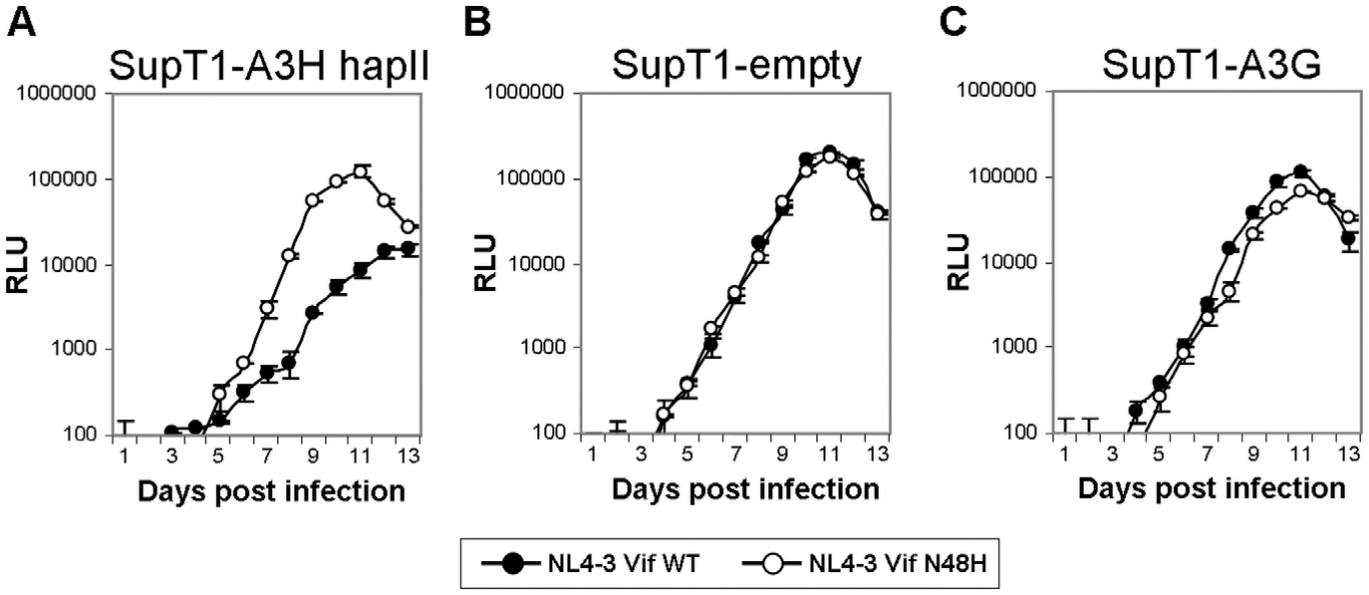
NL4-3 Vif 48H replicates more efficiently on SupT1 cells expressing A3H-hapII. NL4-3 WT (closed circles) and NL4-3 Vif N48H (open circles) were used to infect SupT1 T-cell lines expressing untagged APOBEC3H hapII (A3H hapII, **A**), HA-tagged APOBEC3G (A3G, **B**), and the empty vector (**C**). Clarified supernatants were harvested each day and infectivity was determined on TZM-bl reporter cells. Representative infections of each cell lines are shown. Error bars represent standard deviations of triplicate TZM-bl infections.

Vif position 48 is specific for A3H recognition in single cycle infectivity assays in which APOBEC3 is expressed to high levels in the HEK 293T producer cells. To asses the impact of Vif position 48 on NL4-3 replication in a more physiological experimental system, we infected previously described SupT1 T-cell lines that express low levels of A3G or A3H hapII [16] with NL4-3 WT and NL4-3 Vif N48H. NL4-3 WT replication was delayed by an order of magnitude on SupT1 cells expressing A3H hapII (Figure 7A) indicating that NL4-3 WT fails to efficiently counteract A3H when expressed at near physiological levels in T cells [16]. However, the N48H mutation in Vif improved viral replication to levels observed with the empty vector and A3G expressing cells, indicating that N48H also efficiently counteracts A3H in the setting of spreading infections. Of note, both viruses replicated with similar efficiencies on cells expressing the control empty vector (Figure 7B). Interestingly, the replication of NL4-3 WT was slightly improved compared to the N48H mutant on A3G expressing SupT1 cells, which may indicate that a Vif encoding 48N is somewhat better adapted to counteract A3G (Figure 7B). Taken together, the data indicate that Vif position 48 is specific for counteracting A3H in both single cycle as well as multiple round infection models.

## Discussion

Over the past years, a series of publications established that some human A3H proteins (e.g., A3H hapII) exert potent activity against HIV-1, however, conflicting data regarding the sensitivity of A3H-hapII to HIV-1 Vif remain. Here we show that A3H-hapII is resistant to NL4-3 Vif but sensitive to LAI Vif, which reconciles the published findings based on which Vif was used in the respective studies. The difference in Vif activity against A3H-hapII is likely caused by its ability to interact with A3H-hapII as shown by co-immunoprecipitation (Figure 4). We pinpointed the difference in A3H-hapII degradation activity between NL4-3 and LAI Vif to a critical Vif residue at position 48 (Figure 5). Our group recently reported the importance of this specific Vif residue in combination with Vif position 39 (especially for HIV-1 subtype F Vifs) to efficiently degrade A3H-hapII [17]. NL4-3 and LAI both already contain the active phenylalanine at position 39, making the amino acid identity at position 48 the sole determinant for A3H-hapII neutralization in the context of these two commonly used subtype B molecular clones.

In addition, we show that the A3H RED variant is sensitive to both LAI and NL4-3 Vif [20], pointing to D121E as an molecular determinant for A3H sensitivity (Figure 3). Although this variant has not been found in cellular transcripts in the human populations, chimpanzees and the reconstructed human ancestral A3H encode a glutamic Acid (E) at position 121 [11]. We speculate that the E121D change in A3H-hapII in modern humans may be beneficial, as it would protect from a broader spectrum of HIV-1 strains similar to NL4-3.

Two A3H variants encoding RKE and RKD showed a strong restriction of HIV-1 and were resistant to both Vifs tested (Figure 3B). Interestingly, the A3H RKE genotype variant was detected in at least one Caucasian individual [14], suggesting that some humans may encode A3H variants that are both active against and resistant to HIV-1.

A recent study showed that the A3H-hapII genotype correlates with decreased plasma viremia in HIV-1 infected individuals indicating a role of A3H in HIV-1 disease progression [23]. In addition, a study that included Vifs from HIV-1 infected individuals with different A3H haplotypes indicated that a HIV-1 infected patient homozygous for A3H-hapII harbored Vif variants that were more efficient in counteracting A3H-hapII compared to patients heterozygous or homozygous for the inactive A3H-hapI [18]. These observations point to the possibility that an HIV patient’s Vif adapts to degrading active A3H-hapII, but not inactive A3H proteins since the later variants fail to exert selection pressure since their neutralization is not required for efficient replication. This notion is further supported by our previous study, which showed that a large panel of Vifs alleles derived from different HIV-1 subtypes degraded A3G and A3F efficiently, whereas only half of these Vifs variants could counteract A3H-hapII [17]. Vif protein sequence analysis of 1,286 HIV-1 subtype B Vifs (HIV Los Alamos database, premade Vif protein alignment) shows that position 48 either encodes a histine (H, 71.5%) or an aspargine (N, 28.3%). Thus, while the majority of subtype B Vifs likely display activity against A3H hapII there remains a substantial number of subtype B Vif variants that lacks specific activity to degrade A3H.

The amino acid identity at Vif position 48 specifically affects A3H degradation, whereas A3D, A3F and A3G degradation in single cycle infectivity assays remained unchanged (Figure 6A and 6B). Testing the specificity of NL4-3 Vif 48N and 48H in multiple round infection experiments in T cell lines showed that replication is identical in the absence of APOBEC3 expression (Figure 6A). A sizable, 10-fold reduction in replication is observed on SupT1 cells expressing A3H hapII (Figure 6C), underscoring that the results observed in single cycle infectivity assays are well recapitulated in spreading infections. Interestingly, we observed a small replication delay for NL4-3 48H on cells expressing A3G (Figure 6B), which may indicate that viruses that are better adapted to counteracting A3H-hapII may have lost some activity against A3G.

Collectively, these data suggest a scenario in which the role of A3H on HIV-1 disease progression is both affected by the patients’ own A3H repertoire, but also by the ability of the HIV-1 Vif protein to counteract the respective A3H variants. Future studies are required to elucidate the role of A3H on HIV-1 replication in the context of different subtypes as well as on HIV-1 disease and AIDS progression.

## Materials and Methods

### Plasmids

The replication-competent molecular clones NL4-3 [24] and NL4-3 ΔVif [25] and LAI [26] were provided by the AIDS Research and Reference Reagent Program, Division of AIDS, NIAID, National Institutes of Health NIH Reagent Program [25].

The LAI ΔVif molecular clone was constructed by deleting the NdeI-StuI fragment resulting in the deletion of 284 nucleotides in the *vif* open reading frame. NL4-3 N48H and LAI H48N were constructed using standard overlap PCR mutagenesis.

The mammalian expression plasmids pTR600 containing amino-terminally FLAG-tagged A3H hapI-GKE, A3H hapII-RDD, the six site-directed A3H mutants and A3G were described previously [12]. The A3H RED and Vif mutants were constructed using standard overlap PCR mutagenesis, as previously described, using A3H hapII-RDD and NL4-3 and LAI Vifs as template, respectively [12]. All primer sequences are available upon request.

NL4–3 Vif and LAI Vif proteins were expressed using pCRV1 as described previously [27].

### Culture of Cell Lines

HEK 293T and TZM-bl reporter cells were maintained in Dulbecco's modified Eagle medium (DMEM) supplemented with 10% fetal bovine serum (FBS) and 100 U/ml penicillin-streptomycin. TZM-bl cells were provided by the AIDS Research and Reference Reagent Program, Division of AIDS, NIAID, National Institutes of Health NIH Reagent Program [28].

SupT1 T-cells expressing empty vector, 3xHA tagged A3G and untagged A3H hapII were kindly provided by Dr. R. Harris [16]. SupT1 cells were maintained in RPMI medium supplemented with 10% fetal bovine serum (FBS), 100 U/ml penicillin-streptomycin and 0.5 mg/ml G418 (Mediatech).

### Vif-mediated A3H and A3G Degradation

The FLAG-tagged A3H-hapII and A3G expression vectors (100 ng) were co-transfected with increasing amounts of NL4-3 or LAI Vif pCRV1 expression plasmids (0, 2.5, 5, 10, 25 and 50 ng) and pCRV1 empty plasmid (total amount of pCRV1 50 ng). The transfections were performed in a 24-well format using 4 µg/ml polyethylenimine (PEI; Polysciences, Inc.). Transfected cells were lysed two days post-transfection in 1% sodium dodecyl sulfate (SDS), 50 mM Tris-HCl (pH 8.0), 150 mM NaCl, and 5 mM EDTA. Five µl of 4x lithium dodecyl sulfate (LDS) sample buffer (NuPAGE; Invitrogen) and 2 µl of sample reducing agent (NuPAGE; Invitrogen) were added to 13 µl of the lysate and heated for an additional 10 min at 70°C. Proteins were separated on 10% SDS-polyacrylamide gels (Invitrogen), transferred onto polyvinylidene difluoride (PVDF) membranes (Pierce), and probed with anti-FLAG M2 monoclonal antibody (Sigma), rabbit polyclonal Vif antiserum (AIDS reagent, catalog number 2221) [29] and anti-GAPDH (glyceraldehyde-3-phosphate dehydrogenase) (Sigma) or anti-Tubulin (Sigma) to ensure equal protein loading. Membranes were subsequently incubated with horseradish peroxidase-conjugated secondary antibodies (Sigma), developed with SuperSignal West Pico (Pierce), and detected by using the Fujifilm Intelligent Lightbox LAS-3000 instrument and Image Reader LAS-3000 software. For quantification, non-saturated signals were background subtracted and FLAG signals without Vif were set at 100%.

### Assesment of Viral Infectivity using Single Cycle Infectivity Assays

The FLAG-tagged A3H-hapII and A3G expression vectors (0, 25, 50 and 100 ng) were co-transfected with the different HIV molecular clones, NL4-3 WT, NL4-3 ΔVif, LAI and LAI ΔVif (500 ng) in 293T cells. For the A3H variants 20 ng was co-transfected with the different HIV molecular clones (500 ng). The transfections were performed in a 24-well format using 4 µg/ml polyethylenimine (PEI; Polysciences, Inc.). The culture medium was replenished after 24 h and the supernatants were harvested 48 h after transfection, clarified by centrifugation, and used to infect TZM-bl reporter cells. TZM-bl cells were infected in triplicate with 20 µl of cell-free viral supernatants in 96-well plates. Beta-galactosidase activity was quantified 48 h after infection by using chemiluminescent substrate (Tropix; Perkin-Elmer), as previously described [12]. The data from three independent transfections were used to calculate average values and standard deviations.

### Assesment of Viral Replication in Spreading Infection Experiments

Viral stocks were generated by transfecting HEK 293T cells with 500 ng of NL4-3 WT and NL4-3 N48H molecular clones in 24-well plates using 4 µg/ml polyethylenimine (PEI; Polysciences, Inc.). Supernatants were harvested 2 days post transfection, clarified by centrifugation, filtered and aliquots stored at −80°C until further use. Infectivity titers of the viral stocks were determined by infecting TZM-bl reporter cells in triplicate with serial dilutions of each virus as previously described [30].

1.5×10*6 cells of each SupT1 cell line (empty, A3G, A3H) were infected (MOI 0.5) in a 24-well format (1.5 ml) and washed with PBS 10 hours post infection. Every day over a two week period, culture supernatants (200 µl) were collected, clarified and stored at −80°C. Cultures were supplemented with fresh media each day (250 µl). At the end of the infection, the cryo-preserved culture supernatants were used to infected TZM-bl cells in triplicate (10 µl, 96-well plates). Beta-galactosidase activity was quantified 48 h after infection by using chemiluminescent substrate (Tropix; Perkin-Elmer), as previously described [12]. Two independent infections of SupT1 cell lines were performed.

### Co-immuno-precipitation

5 µg of 3xHA-tagged A3H-hapII in PTR600 or empty pTR600 were co-transfected with 1 µg of pCRV1-NL4-3 Vif, pCRV1-LAI Vif or empty PCRV1 in 293T cells in 10-cm dishes. Cells were treated with clasto-Lactacystin β-lactone (10 µM, Sigma) for 10 hours and 48 hours post transfection the cells were washed with PBS and lysed in mild lysis buffer (1% Triton X-100 in PBS supplemented with EDTA-free protease inhibitor cocktail, Roche). Lysates were cleared by centrifugation at 8,000×g for 10 minutes and incubated with anti-HA tagged beads (Sigma) for one hour at 4°C. Supernatants were discarded and the beads were washed by incubating with mild lysis buffer for another hour at 4°C. Subsequently, beads were washed 4 times with mild lysis buffer and proteins were eluted by boiling in LDS loading buffer (Sigma). Proteins were analyzed by western blot using anti-HA monoclonal antibody (Sigma), rabbit polyclonal Vif antiserum (AIDS reagent, catalog number 2221) [29] and anti-GAPDH (Sigma).
